# Densification and Mechanical Enhancement of Invasive South African Hardwoods: *Prosopis glandulosa* and *Acacia mearnsii*

**DOI:** 10.3390/ma19050954

**Published:** 2026-03-01

**Authors:** Matin Naghizadeh, Marthie E. Niemand, Ernst H. G. Langner, Aimin S. Sivanda, Karel G. von Eschwege

**Affiliations:** 1 Department of Chemistry, University of the Free State, Bloemfontein 9300, South Africa; naghizadeh.m@ufs.ac.za (M.N.); langneeh@ufs.ac.za (E.H.G.L.); 2 Centre for Environmental Management, University of the Free State, Bloemfontein 9300, South Africa; kempm@ufs.ac.za (M.E.N.); aiminsivanda@gmail.com (A.S.S.)

**Keywords:** invasive species, densified wood, wood strength, low cost, delignify

## Abstract

Wood used in construction varies in density, leading to differences in strength and rigidity. Wood densification has recently emerged as a promising technique to address these limitations and enhance material performance. This study explores the potential of two abundant and low-cost invasive hardwood species in South Africa—*Prosopis glandulosa* (Honey Mesquite) and *Acacia mearnsii* (Black Wattle)—as sources for producing densified wood. A range of strengthening methods, including chemical, pressure, and heat treatments, were applied and compared. After partial delignification and hot pressing, sample thicknesses were reduced by 40% for *Prosopis* and 50% for *Acacia*, yielding substantial increases in flexural strength of 216% (22.61 MPa) for *Prosopis* and 334% (24.65 MPa) for *Acacia*. In addition to anatomical imaging, analyses of lignosulphonate content, and thermogravimetric profiling, the study also evaluated several practical, carpentry-relevant mechanical properties. These included comparative tests for flexural and compressive strength, nailing and sanding performance, as well as assessments of water absorption, electrical resistivity, and flame-holding capacity.

## 1. Introduction

Wood, a material utilized by humans for millennia, remains a popular choice in construction. Due to its low density, its versatility extends to transportation, particularly shipbuilding. Nevertheless, this low density is often linked to a lack of strength and rigidity, which restricts its use in high-stress situations [1,2]. Wood densification recently emerged as a promising solution. To help overcome the limitations of untreated wood, such as inferior mechanical properties and dimensional instability, a process of delignification and mechanical heat-compression was recently reported [3,4]. This treatment process leads to a significant increase in the overall density and strength of the wood.

In general, wood qualities can be readily modified through the use of various techniques, such as hot pressing, steam and/or chemical treatment, and hydrothermal alteration. Low-cost wood densification, achieved through mechanical compression, results in a material that exhibits enhanced strength, hardness, and stiffness. Compressed wood is highly versatile and can be used in various applications, including furniture, flooring, and structural components in building and vehicles [5,6,7]. Utilization of densified wood in such diverse sectors exhibits encouraging outcomes, particularly when used in load-bearing structural elements [8].

A recent study compared the mechanical characteristics of natural and densified cedar, oak, and poplar woods [9]. The findings revealed that natural oak exhibits the highest tensile strength at 115 MPa. Following the densification process, oak’s strength significantly increased to 584 MPa. The fracture values for natural oak and its densified counterpart were measured at 1.8 MJ m^−3^ and 5.3 MJ m^−3^, respectively. In comparison, poplar wood demonstrated a strength of 56 MPa in its natural state, which is less than half that of oak. However, after densification, poplar’s strength significantly improved to 432 MPa. Cedar wood had the lowest natural strength among the three kinds of wood, measuring 47 MPa. Densification of cedar wood resulted in a notable enhancement in strength, reaching 550 MPa. These findings underscore the considerable improvement in the mechanical properties of wood that can be achieved through the process of delignification (DL).

Wang et al. [10] examined the impact of alkali and maleic acid hydrotropic DL on poplar wood. The study focused on the removal of different amounts of lignin and hemicellulose through the DL process. After DL treatment, wood samples were subjected to hot pressing at 180 °C. The researchers then analyzed the changes in the wood’s microstructure, chemical content, and dimensional stability. The results showed that excessive removal of lignin during the DL process negatively affected the dimensional stability of the wood before heat treatment, i.e., removing too much lignin made the wood less dimensionally stable.

The study conducted by Maturana et al. investigated the effectiveness and consistency of a partial DL procedure on three different types of hardwoods: *Carapa guianensis* (Andiroba), *Brosimum utile* (Sande), and *Dipteryx oleifera* (Choiba) [11]. The DL process eliminated a fraction of the lignin and hemicellulose. While there were only minor alterations in wood density after DL, there was a significant decrease in both the modulus of elasticity (MOE) and modulus of rupture (MOR). Previous research has shown that spruce veneers that have been partially delignified and densified through the use of alkalis and organic solvents can exhibit exceptional tensile properties [12]. This process significantly improves the bending strength and modulus of elasticity, resulting in the production of sturdy plywood. This method enhances the mechanical properties of veneers while maintaining interlaminar shear strength. The preservation of wood strength was significantly improved by partially removing lignin, which increased its mesoporosity without causing irreversible damage, a common issue with complete DL [13].

In [14,15], DL and densification of green and dried poplar involved solutions of sodium hydroxide and sodium sulfite at different temperatures for 7 h. Before densification, the wood underwent a washing process and subsequent analysis, resulting in an 80% reduction in thickness. Different temperatures and durations of pressing were employed throughout the process of densification. The bending strength significantly increased, reaching a maximum value of 450 MPa. This improvement was observed under ideal conditions, which involved eliminating approximately 90% of the lignin content and subjecting the material to a pressing time of 24 h at a temperature of 130 °C.

*Prosopis glandulosa* and *Acacia mearnsii* are commonly employed in various applications, such as construction, due to their adaptability and rapid growth rates. Originating from Mexico and the southwestern USA, *Prosopis glandulosa* finds utility in rustic construction, posts, and furniture within its natural habitat and beyond. Both species are prized for their wood, which serves as a fuel source and timber in arid and semi-arid regions [16,17]. However, in South Africa, both these species are classified as invasive, posing a significant threat to native ecosystems by displacing indigenous flora and diminishing biodiversity.

Limited data has been published on the densification of solid wood and its mechanical properties; studies with a focus on alterations in density, strength, hardness, and dimensional stability that occur as a result of the densification procedure have only investigated a few selected wood species [9]. Reduction in swelling as a result of resilience to moisture uptake was also observed [18,19,20,21,22]. Understanding the performance and durability of wood-based buildings relies heavily on the properties of the wood materials. Current and future investigations should aim to provide experimental data to help answer inquiries from the construction sector regarding the practical use of machining techniques and the suitability of densified wood for various applications.

*Prosopis glandulosa*, commonly known as Honey Mesquite, was introduced to South Africa in the late 1880s from South, Central, and North America. While the wood of *Prosopis glandulosa* is known for its hardness [23,24,25], large impenetrable tracts of Prosopis thickets cause loss of valuable pasture in the arid and semi-arid areas in South Africa. The negative impacts of this aggressive invader also include a change in soil chemistry, the soil microbial balance, and soil quality, while eradicating *Prosopis glandulosa* is extremely costly and difficult [26].

Black Wattle, or *Acacia mearnsii*, is an invasive tree species that thrives in South Africa’s various habitats. This Australian evergreen tree has been introduced and cultivated worldwide, including in South Africa, where it is used in ecological studies and industry. *Acacia mearnsii* has extraordinary growth qualities. It grows quickly under suitable conditions. This tree can reach 5–10 m in 5–7 years, presenting a striking shape with a straight and slender trunk. *Acacia mearnsii* wood is distinctive and flexible. A wood density of 670 kg/m^3^ to 850 kg/m^3^ makes this species highly valued in industrial and commercial industries [27,28,29,30,31].

Despite their invasive tendencies, these species’ economic and versatile attributes continue to drive their utilization [27,32]. To gain a deeper understanding of the capabilities of enhanced natural materials, we conducted a comprehensive comparative analysis of wood samples that were treated in different ways. Analyses were performed on all samples, encompassing their flexural and compressive strengths, resistance to nailing and sanding, moisture absorption and flammability, thermal stability, and electrical conductivity/resistance. The research findings presented here offer useful insights into the diverse characteristics and serve as a foundation for future novel uses of cost-effective, improved wood.

## 2. Experimental Section

### 2.1. Plant Material Collection and Identification

The wood samples of *Prosopis glandulosa* (Honey Mesquite) and *Acacia mearnsii* (Black Wattle) used in this study were collected from invasion sites in South Africa. *Prosopis glandulosa* wood was supplied by Dr. C. P. Cronje (Zoning & Permissions Advisor) from the Prieska area in the Northern Cape Province (GPS coordinates: −29.808780 S, 22.714670 E). *Acacia mearnsii* wood was supplied by NTE (https://nte.co.za/, accessed on 13 February 2026) and was collected from Ptermaritzburg area in the KwaZulu-Natal Province (GPS coordinates: −29.0203132 S, 30.7789173 E).

Formal species identification was confirmed by co-author Marthie E. Niemand, who is affiliated with the Centre for Environmental Management at the University of the Free State, Bloemfontein, South Africa.

Both *Prosopis glandulosa* and *Acacia mearnsii* are classified as invasive alien plant species in South Africa under the National Environmental Management Biodiversity Act, 2004 (NEMBA). *Acacia mearnsii* is listed as a Category 2 invasive species, and *Prosopis glandulosa* is listed as Category 1b (in Eastern Cape, Free State, North-West, and Western Cape) and Category 3 (in Northern Cape) according to the Alien and Invasive Species (AIS) Regulations under NEMBA. Under these regulations, landowners are legally obliged to manage and control the spread of these species. The collection of wood samples for research and commercial purposes complies with the requirements of NEMBA and the Conservation of Agricultural Resources Act, 1983 (CARA), and is consistent with the IUCN Policy Statement on Research Involving Species at Risk of Extinction and the Convention on the Trade in Endangered Species of Wild Fauna and Flora. No specific collection permits were required as the removal of invasive plant material contributes to mandated control efforts.

Due to the invasive nature of these species and the focus of this study on wood material properties rather than botanical taxonomy, voucher specimens were not deposited in a herbarium. Both species are widespread across more than 100,000 square kilometers in South Africa, and extensive documentation of their distribution and characteristics is available in the scientific literature.

### 2.2. Chemicals and Instruments

The delignification process of wood from two species, *Prosopis glandulosa* from the Northern Cape Province and *Acacia mearnsii* from the KwaZulu-Natal Province, involved the use of sodium hydroxide (NaOH, >97%, Merck, Rahway, NJ, USA), sodium sulfite (Na_2_SO_3_, >98%, Merck), and distilled water.

**Figure 1:**
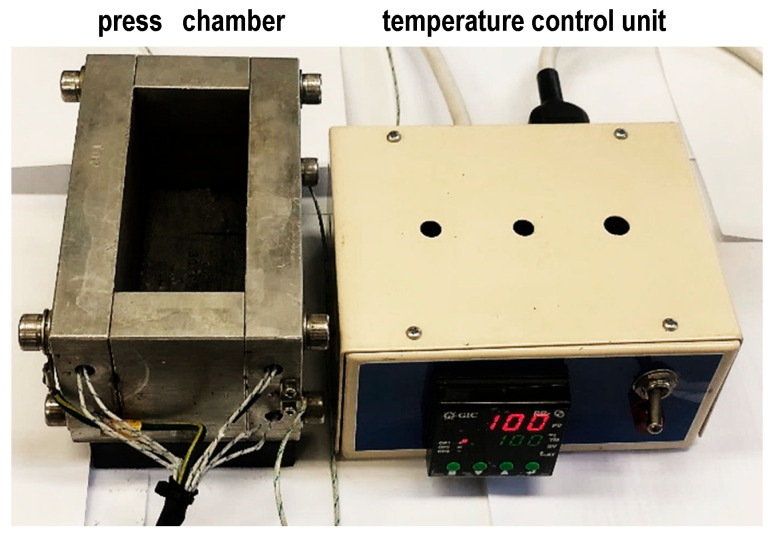
Temperature-controlled press chamber.

A 6-ton mechanical press fitted with a 2500 kg load cell (Adam Equipment, UK, type GK Indicator Platform scale) and a temperature-controlled press chamber (inner dimensions: 100 × 45 × 45 mm), which was made in-house, was employed for wood densification (Figure 1).

The same equipment was used for measuring the flexural and compressive strengths, as well as the force required to press a nail through the wood samples. Small mechanical devices required for the various tests were manufactured in-house. The sanding test was conducted using a belt sander, with a sanding belt speed of 250 m per minute and P120 grit aluminum oxide sandpaper. A UNI-T, China, UT513A insulation resistance tester (Dongguan, China) was used to measure the electrical resistivity of all wood and reference samples (1 mm thickness), up to 5000 V. A METTLER TOLEDO, Switzerland, TGA/SDTA851 analyzer (Greifensee, Switzerland) was used to conduct thermogravimetric (TGA) analyses under ambient conditions. TGA data were analyzed using METTLER STARe Evaluation software 9.01. A Bernzomatic^®^ UL100 propane torch (Chilton, WI, USA), with a flame temperature of ca 1980 °C in air, was used for the flame tests. A Carl Zeiss Microscopy GmbH, Germany, ZEISS Stereo Discovery V8 optical microscope was used for imaging the microscopic cell structures of the different wood samples. Safranin O (Sigma-Aldrich, St. Louis, MO, USA) was used for staining the lignin for microscopic analysis.

### 2.3. Sample Treatment

To partially delignify the wood, samples with dimensions of 95 × 25 × 25 mm were submerged in boiling solutions containing NaOH (50 g, 2.5 M) and Na_2_SO_3_ (25 g, 0.4 M) for 40 and 80 min periods [33]. Subsequently, the wood blocks were washed (20 min) twice in boiling water (1 L) to remove residual chemicals.

For densification, all samples were subjected to a pressure of 5 MPa at a temperature of 100 °C. An untreated sample of each wood species was also cold-pressed at room temperature for comparison. The compression times were 3 h for cold pressing, 5 h for hot pressing, and 12 h for the samples that underwent the delignification process.

### 2.4. Tests and Analyses

**Figure 2:**
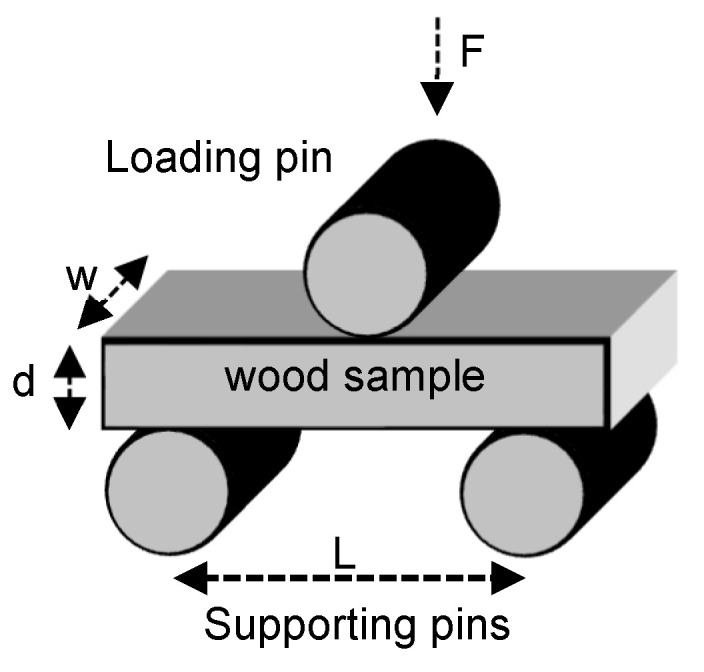
Setup for three-point flexural strength test.

*Flexural strength* was assessed through the implementation of the three-point test, wherein a load is exerted at the midpoint of a supported sample until it fractures (Figure 2) [34,35].

Flexural strength (σ) was determined using the formula:

σ = 3FL/2wd^2^

where F represents the maximum force applied in Newtons, L is the distance between two supporting pins, w denotes the width of the sample, and d is the depth/thickness of the sample, all in meters. Processed samples of different thicknesses were subjected to a compressive force, which was applied perpendicular to the wood grain in the transverse direction.

*Compressive strength* (P, in MPa) was determined using the formula:

P = F/A

where F represents the highest load (or load until failure) applied to the material (in Newtons) and A is the cross-sectional area of the sample that withstands the load. To measure the compressive strength, the dimensions of all wood samples were adjusted to form cubes with a side length of 10 mm. The samples were placed between two flat steel plates and subjected to compression. The maximum force exerted on the samples, just before collapse, was measured. The force was applied in the same direction as the wood grain (longitudinal direction).

*Nail resistance* tests were conducted similarly to compression testing. A hardened, sharp steel nail measuring 20 mm in length and 2 mm in thickness was affixed to the press. The nail was then driven into the center of each wooden sample (transverse direction), which had dimensions of 10 × 10 mm and 15 mm in length. The maximum force exerted during this process was documented.

*Abrasion resistance* or sanding tests were performed by using a belt sander on a 1 cm^2^ pre-weighed sample for a period of 5 s [36]. The weight of the belt sander was 2.94 kg, which exerted a constant pressure of 0.29 MPa on the samples. After the sanding, the samples were weighed again, and the percentage mass loss was calculated and recorded.

For the *moisture uptake* tests, wood samples measuring 10 × 10 × 5 mm were dried in an oven at 110 °C for 24 h and then weighed at room temperature. The dried specimens were then immersed in water for 24 h at ambient temperature. After immersion, the moist samples were dried using a paper towel and subjected to atmospheric drying for 20 min before being re-weighed. The mass gain was transformed into percentage water absorption [37].

*Thermal stabilities* of all samples were assessed using TGA. The samples were subjected to controlled heating at a rate of 10 °C per minute until they reached a temperature of 600 °C in the presence of ambient air [38].

*Flame-holding* tests on wood samples measuring 20 × 10 × 10 mm were subjected to a direct gas flame for 15 s. After the torch was removed, the sample’s ability to sustain a flame was assessed by documenting the duration it took for the flame to die out on its own.

The *lignosulfonate content* of selected samples was quantitatively determined through the UV–vis spectrophotometry methods of Hon and Dence [39,40]. Specifically, the absorbances of slightly acidic aqueous solutions of the lignin samples were measured in the 210–400 nm wavelength range. Lignin content was then quantified as a mass percentage relative to the solid samples, with the results referring specifically to the lignosulfonate fraction.

An *optical microscope* was utilized to assess the microscopic anatomical properties of both *Prosopis glandulosa* and *Acacia mearnsii* species, both before and after treatment, but not from the same pieces of wood. A WSL sliding microtome was used to prepare thin sections from the transverse plane of each sample to compare the general anatomical structure of both treated and untreated wood. Each thin section was cut to between 12 and 14 µm in thickness, and stained using a 1% Safranin O staining solution, following the standard approach reported by Ruzin [41]. The lignin in the cell walls, which had become stained, exhibited a vivid red to deep pink color, thereby accentuating the observable alteration in cell structure prior to and following treatment.

*Electrical resistivity* measurements, including breakthrough voltage, were conducted on samples of *Prosopis glandulosa* and *Acacia mearnsii* species. The measurements were taken under three different conditions:

- As is, under ambient conditions;
- After drying the samples for 2 h at 120 °C;
- After saturating the samples in furniture oil (raw linseed oil) and wiping them dry.

Measurements were also conducted on reference materials using meter settings of 500, 1000, 2500, and 5000 volts, all within the permissible range.

## 3. Results and Discussion

**Figure 3:**
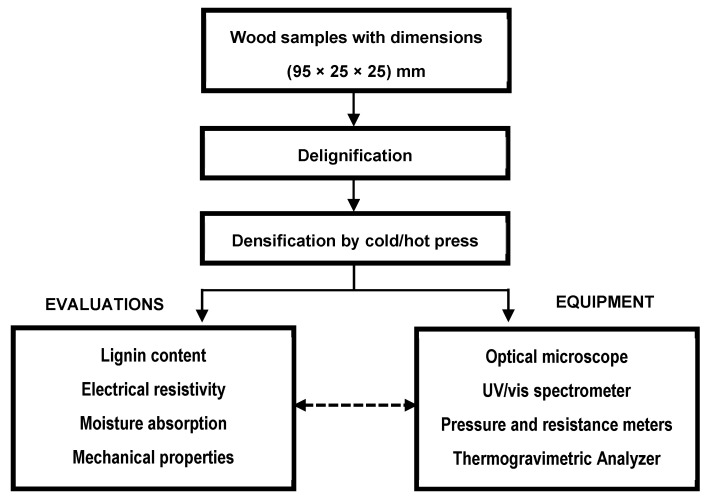
Experiment design.

Figure 3 depicts the experimental arrangement used in this study. All samples were uniformly resized as specified. The heating chamber’s cavity size, as shown in the photo in Section 2.1, was larger than that of the samples. This allowed for lateral expansion and also provided space for moisture evaporation under compression at a high temperature of 100 °C.

In contrast to previously reported open-compression platforms [42,43,44,45], we constructed a sealed press chamber to ensure rapid, homogeneous heating and temperature regulation. Water vapor was able to exit through small apertures located on the sidewalls. The compression periods for samples that were not boiled or delignified were determined based on the duration required for the applied mechanical pressure to reach a stable state, i.e., where it stops decreasing. Untreated wood achieved pressure stability after around 2 h when pressed at room temperature. However, when pressed at 100 °C, it took up to 4 h for the pressure to stabilize. The extended duration can be attributed to the loss of moisture and chemical reconfiguration at higher temperatures. In both situations, an extra hour was included, resulting in press times of 3 h for cold pressing and 5 h for hot pressing. The wet DL samples were boiled for 40 and 80 min and promptly hot-pressed after being washed twice in clean boiling water. In this case, a prolonged compression time of 12 h was required to achieve fully dried and densified wood. Pressing the wood for a short duration in these situations resulted in samples that were swollen in the center due to the presence of residual water trapped inside the wood fibers. Furthermore, reducing the temperature to below 100 °C considerably decelerated the wood drying process.

In previously reported studies, researchers focused on removing only a portion (40%) of the lignin before compression, with long delignification boiling times of up to 7 h [9]. Our initial testing showed that shorter boiling times are sufficient, which is crucial because it can lead to significant cost savings in terms of energy consumption during large-scale production. Consequently, lignin extraction periods were set to 40 and 80 min. Subsequent investigations and comparisons of different wood species that have potential economic uses were conducted under the same conditions outlined in this paper. The objective was to reduce production time, as well as chemical and energy expenses throughout the entire process, which is necessary for developing the foundation for a sustainable business model. In addition to reducing the drying time of the delignified samples, it was also considered important to report comparisons with other more affordable treatments, such as direct cold pressing of untreated wood, hot pressing, and water-boiling followed by pressing—all without delignification.

Lignin content, as determined by UV/vis spectrophotometry, was associated with mass reductions of 13% and 18% for the 40 min and 80 min *Prosopis glandulosa* DL procedures, respectively. The lignin content of other *Prosopis* species, such as *Prosopis laevigata*, has been reported to range from 29.8% to 31.4% [46,47]. Typically, tropical hardwood species have lignin levels that range from 10 to 40% [48]. The analytical data for the extractions of *Acacia mearnsii* showed mass reductions of 23% and 25%, respectively. The results are consistent with the lignin contents that have been reported for many *Acacia* species, which range from 21 to 25% [49].

**Figure 4:**
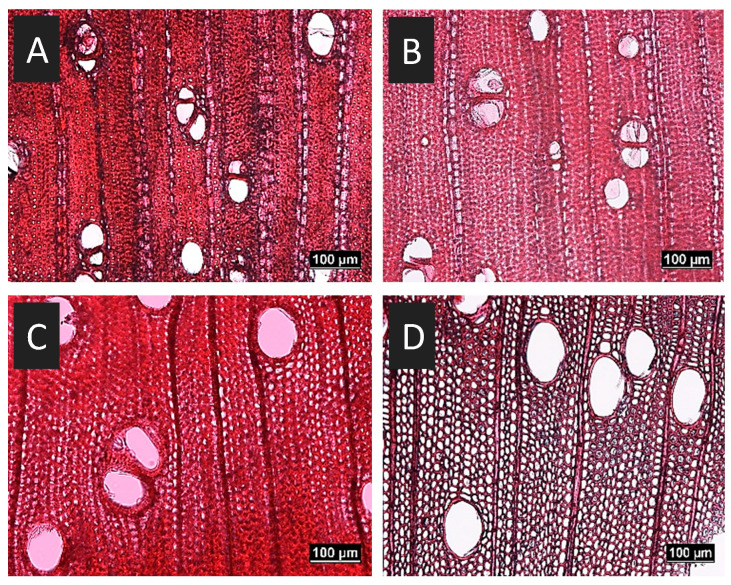
Optical microscopy images of cell structures in untreated (**A**,**C**), and 80 min delignified (**B**,**D**) *Prosopis glandulosa* (**top**) and *Acacia mearnsii* (**bottom**) wood samples.

The microscopic morphologies of the untreated (left) and delignified (right) wood samples are shown in Figure 4. The diffuse porous wood of both *Acacia mearnsii* and *Prosopis glandulosa* appears to be mostly intact before and after treatment, except for a slight reduction in lignification in the treated samples. The fibers of both species are thick-walled, adding to the wood strength of these species and therefore, less distortion of the cells was expected. However, after treatment, the fibers of both exotic species exhibited thinner cell walls with larger visible lumina, but without any change in shape, which was again expected.

*Acacia mearnsii* has solitary vessel elements of similar diameters dispersed throughout the ring structure, which showed no noticeable distortion after treatment. Although the ray cells are intact after treatment, *Prosopos glandulosa* has larger, more pronounced rays than *Acacia mearnsii*. The rays can be described as 2–3 seriate.

These two species are angiosperms, which are much stronger due to their fibers and vessel elements that form part of the wood structure compared to, for example, pines, which are gymnosperms that do not have fibers or vessel elements in their wood anatomy. Their wood structure consists mainly of tracheids and rays. Thus, less distortion is expected in *Acacia mearnsii* and *Prosopis glandulosa* than in pines.

**Figure 5:**
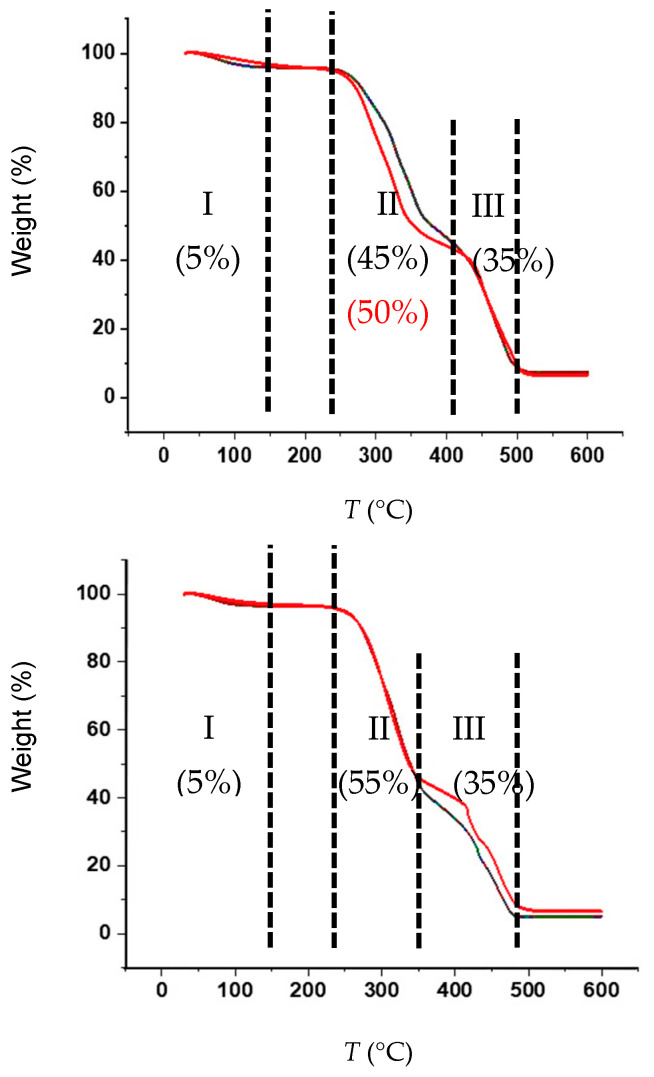
TGA curves of *Prosopis glandulosa* (**top**) and *Acacia mearnsii* (**bottom**) wood samples. (**─**) untreated, (**─**) 80 min delignified. Mass loss percentages are indicated in brackets.

TGA analyses (Figure 5) indicated that the thermal deterioration of both untreated and delignified wood samples can similarly be divided into three distinct stages of mass loss.

- The first phase (I), occurring between room temperature and 150 °C, is attributed to the liberation of approximately 5% water for all samples—this is the only phase that is relevant to the densification process.
- Phase I is followed by significant mass loss (Phase II), starting at 240 °C, which is the result of decomposition of lignin components in several stages. Thermal decomposition of *Prosopis* samples concludes at approximately 410 °C, while for *Acacia*, it concludes at around 350 °C. The mass loss of delignified *Prosopis* was approximately 50%, nearly identical to the 45% mass loss of the untreated sample, likely due to significant densification. The delignified *Acacia* sample, with 50% densification, did not exhibit a substantially different mass loss compared to the untreated sample, both around 55% during Phase II.
- The final phase (III) for *Prosopis* involves rapid degradation after 420 °C, concluding at around 500 °C. For *Acacia*, this step begins at a much lower temperature, 350 °C, and is characterized by fast degradation ending at approximately 480 °C. This final step corresponds to the breakdown of the cellulose structure.

Comparing the TGA curves at higher temperatures, the untreated *Acacia* samples showed an upward shift compared to the 80 min treated samples, while the *Prosopis* species exhibits almost no upward shift, aligning with its inherently denser nature.

**Figure 6:**
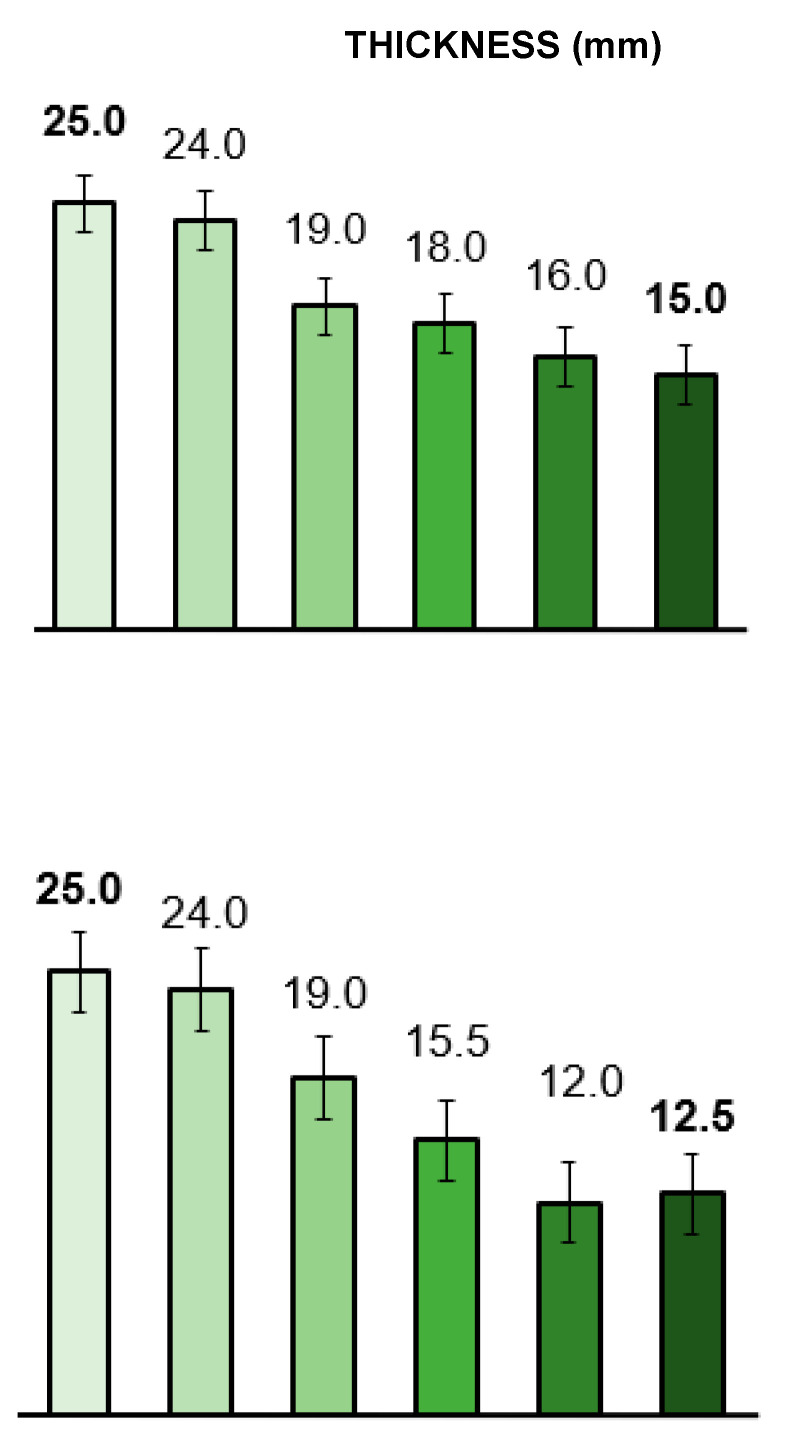
Thickness comparison of *Prosopis glandulosa* (**top**) and *Acacia mearnsii* (**bottom**) wood samples that were ■—untreated, ■—cold-pressed, ■—hot-pressed (100 °C), ■—boiled in water then hot-pressed, ■—delignified for 40 min and hot-pressed, and ■—delignified for 80 min and hot-pressed. Reported values are the means of two experiments.

Figure 6 shows the decrease in thickness (from the original 25 mm) of the wood samples subjected to different treatments. The distinction between the dense *Prosopis glandulosa* (top) and *Acacia mearnsii* (bottom), which is almost half the weight (see Supporting Information Table S2), is clearly apparent. The 80 min delignified samples consistently reached the smallest thickness dimensions under compression. Specifically, the 80 min delignified *Prosopis* was compressed to 60% of its original thickness under 5 MPa of pressure, while *Acacia* was compressed to 50%. This difference in compressibility is attributed to the more permeable and lighter structure of *Acacia*, as observed in the microscope images (Figure 4C,D). The impact of DL is evident, with the 80 min delignified sample resulting in the most compacted material. The most significant reduction in thickness, from 24 to 19 mm, is observed when transitioning from cold to hot pressing for both species. This represents a 24% reduction in original thickness. Both *Prosopis* and *Acacia* exhibited substantial mass loss, as indicated in SI Table S2. The overall mass reduction using hot pressing alone (without prior DL) is likely due to the loss of water and leaching of liquefied organic components during the extended 5 h hot-compression period (see SI Table S1).

**Figure 7:**
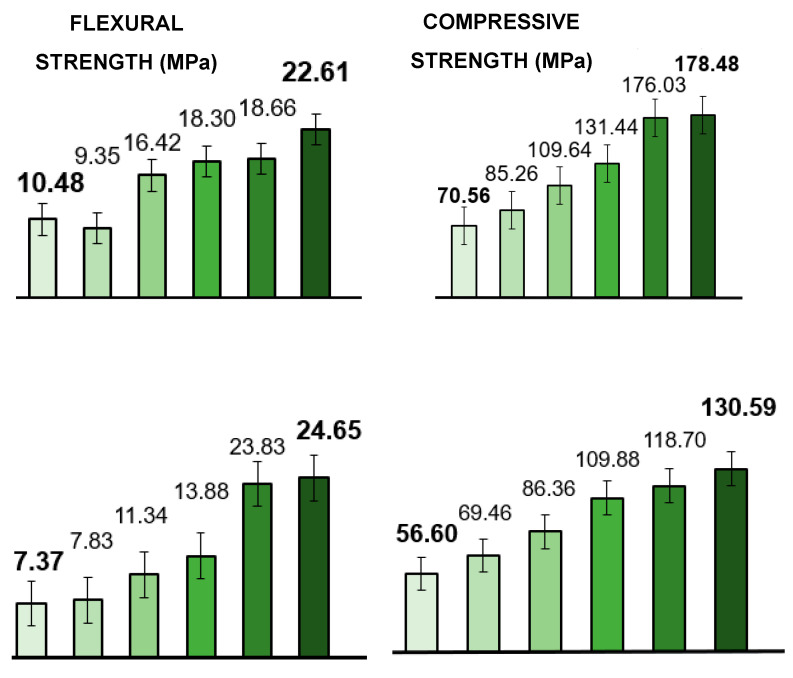
Flexural (**left**) and compressive (**right**) strengths, calculated from maximum pressures measured on *Prosopis glandulosa* (**top**) and *Acacia mearnsii* (**bottom**) wood samples that were ■—untreated, ■—cold-pressed, ■—hot-pressed (100 °C), ■—boiled in water then hot-pressed, ■—delignified for 40 min and hot-pressed, and ■—delignified for 80 min and hot-pressed. Reported values are the means of two experiments.

Figure 7 (left) compares the flexural strengths determined based on the points of maximum pressure just before the point of fracture. The flexural strength data is derived from compressive forces applied perpendicular to the wood’s growth direction, in contrast to compressive strengths. The *Acacia* 80 min delignified sample exhibited a 334% increase in flexural strength, reaching 24.65 MPa. This finding aligns with the research conducted by Song et al. [9], which found that partially delignified materials ended up stronger compared to samples where lignin was completely removed. This is consistent with the measured lignosulphonate content in the 80 min DL sample, as pointed out here earlier. In comparison to the reduction in thickness at a pressure of 5 MPa, the *Prosopis* sample showed a 216% increase, reaching 22.61 MPa.

During compressive strength measurements, increasing pressure was applied along the growth direction of the wood samples until the point of collapse. Smaller samples of a similar size (10 mm cubes) for subsequent testing were all cut from the two original larger samples that were prepared under each process. The maximum pressures/compressive strengths were recorded and are presented in Figure 7 (right). The compressive strength increased by approximately 150% for *Prosopis* and 130% for *Acacia* after 80 min of delignification and hot pressing. These results are consistent with related flexural strength trends. Again, the results for both the flexural and compressive strength measurements showed that the 80 min partial DL with the hot press approach increased the wood strength the most.

Densified and strengthened wood from partially delignified *Acacia mearnsii* shows greater thickness reduction and strength gains during heat-compression compared to *Prosopis glandulosa*, primarily due to intrinsic wood properties. *Acacia mearnsii* typically has a higher initial lignin content (around 25–30% vs. *Prosopis glandulosa*’s 20–25%), facilitating more effective delignification and cell wall collapse under pressure as lignin acts as a rigid matrix that, when partially removed, allows for denser hydrogen bonding between cellulose microfibrils. Its fiber structure features longer, finer fibers with a more uniform helical arrangement and higher microfibril angle flexibility, promoting superior compressibility and plasticization during hot-pressing, unlike the coarser, more irregular fibers and vessel-rich microstructure of *Prosopis glandulosa*, which resist deformation and lead to less efficient packing. Additionally, *Acacia mearnsii*’s lower initial density (0.55–0.85 g/cm^3^) and higher extractive content (up to 25%) enable greater relative densification (up to 80% thickness reduction) and enhanced mechanical interlocking post-compression, yielding tensile strengths exceeding 400 MPa, while *Prosopis glandulosa*’s denser starting matrix (often >0.69 g/cm^3^) and silica inclusions limit shrinkage to ~50–60% and cap strength improvements. These differences underscore *Acacia mearnsii*’s microstructural advantages for optimized densification outcomes [50,51,52,53,54,55,56].

The authors have chosen to investigate and present data from various less costly densification processes as the business case for viable industrial mass production will be heavily dependent on production time, energy and water consumption, as well as the use of chemicals, together with costs associated with chemical waste disposal, etc. From both the thickness reduction and strength data, it is clear that prior water boiling followed by hot pressing most closely approaches the results obtained from costly chemical delignification processes.

**Figure 8:**
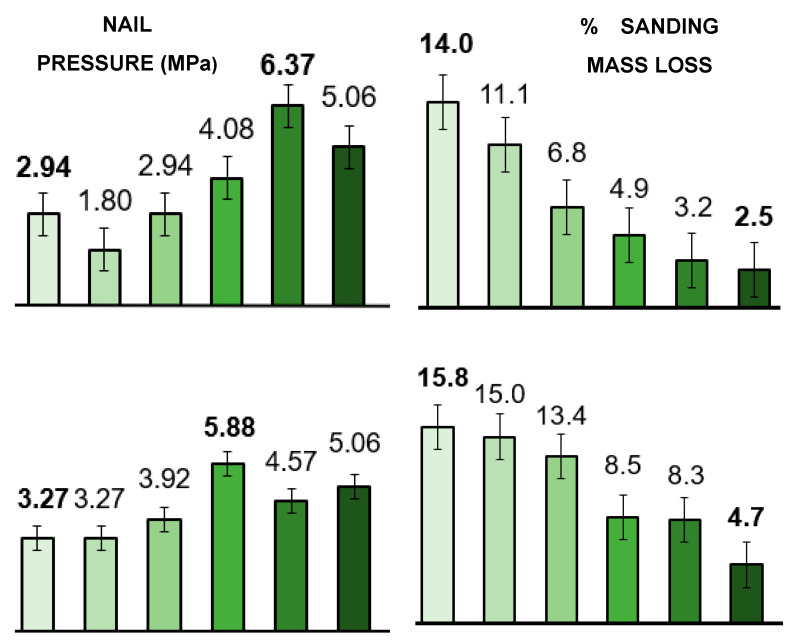
Nail and sanding resistances measured for the *Prosopis glandulosa* (**top**) and *Acacia mearnsii* (**bottom**) wood samples that were ■—untreated, ■—cold-pressed, ■—hot-pressed (100 °C), ■—boiled in water then hot-pressed, ■—delignified for 40 min and hot-pressed, and ■—delignified for 80 min and hot-pressed. Reported values are the means of two experiments.

Carpenters raised questions about the suitability of densified wood for everyday woodwork machining. To test and quantify some of these applications, methods were devised to assess the resistance of densified wood to nailing and sanding, as described in the preceding Experimental section. Regarding nail resistance, a 2 mm thick steel nail was pressed into different samples of similar sizes, and the point with highest pressure was recorded (see Figure 8 (left)). The observations closely matched the flexural strength trends seen for both samples. The pressure required to drive the nail into densified *Prosopis* increased by 120% (6.37 MPa compared to 2.94 MPa for untreated wood). Small/partial to no cracking was observed for the *Prosopis* samples (see SI Table S3). Despite the small size of the samples (10 × 10 × 15 mm), the appearance of tiny cracks directly opposite each other around the relatively thick nail indicated that there was no significant resistance to driving nails into compressed wood. The observations for *Acacia*, however, differed—its samples exhibited random cracking, with some showing full cracks while others showed none. This clearly demonstrates that the behavior depends not only on the wood species but also on variations within individual samples. Therefore, it was deemed important to present the data exactly as recorded, without bias, to accurately reflect the conditions likely to be encountered in large-scale industrial applications.

To simulate practical carpentry working conditions during the abrasion/sanding test, the weight (2.94 kg) of a running belt sander itself was used to apply constant pressure to wooden blocks with an identical surface area. The weight loss of the blocks was measured after sanding for 5 s, see Figure 8 (right). As expected, untreated samples with lower densities exhibited the highest mass loss. The untreated Prosopis sample showed 14% mass loss compared to 2.5% for the densified sample, corresponding to an 82% reduction in material loss, with sanding accounting for only 2.5%. Similarly, the untreated *Acacia* sample had a mass loss that was 334% greater than the most dense sample.

Clearly, densified wood may still readily be nailed and sanded, but nailing and sanding are systematically more difficult for denser samples. In addition, during sizing and machining of the different samples, careful consideration was given to performance and relative ease of sawing, planing, and drilling of treated samples. Notably, no material property changes were observed that could prevent any of these machining techniques or hamper them significantly.

In general, the densified samples have a smoother, almost plastic-like appearance and are less likely to create splinters. During flexural stress testing, the natural wood samples slowly developed cracks and splinters while the pressure increased. In contrast, the densified samples experienced abrupt and complete breaking without splintering, accompanied by a distinct loud snap sound, resembling the behavior of hard plastics.

**Figure 9:**
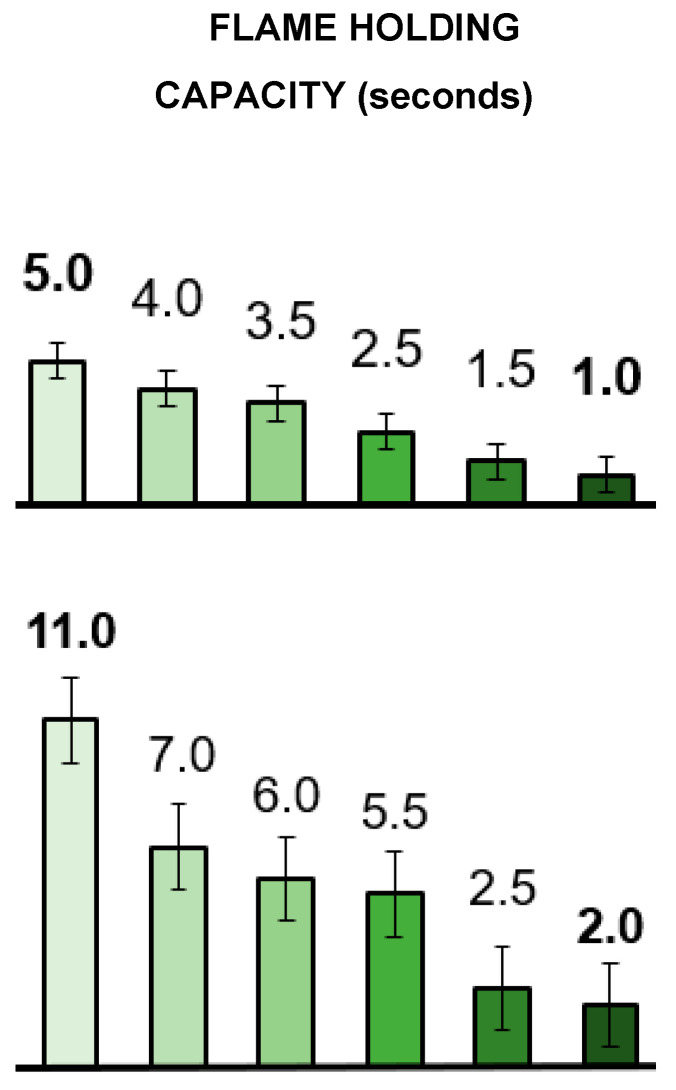
Flame-holding capacity of *Prosopis glandulosa* (**top**) and *Acacia mearnsii* (**bottom**) wood samples that were ■—untreated, ■—cold-pressed, ■—hot-pressed (100 °C), ■—boiled in water then hot-pressed, ■—delignified for 40 min and hot-pressed, and ■—delignified for 80 min and hot-pressed. Reported values are the means of two experiments.

Figure 9 illustrates flame-holding capacities (ability to sustain a flame) of the various samples, i.e., how long after igniting the wood samples with a gas torch before the flames die down. In both species, the downward trends followed corresponding increases in wood densities. For several reasons, this follows the expectation that low-density wood.

- Contains more air spaces, which allow for better oxygen flow;
- Allows heat to penetrate more easily;
- Typically has a larger surface area relative to its mass;

All of these may lead to faster ignition and more rapid and sustained burning.

Densified wood is thus seen to sustain fire less readily, at least directly after initial exposure. This observation is not indicative of other fire properties since it is also known that dense wood tends to burn longer and provide more heat energy over time due to its higher mass and energy content.

Water absorption tests were conducted on all samples of comparable size, and the results were recorded as the percentage increase in mass (refer to SI Table S4). Before immersion, the samples were dried and then placed in water at room temperature for 24 h. Densified samples were found to not exhibit reduced water absorption. This is consistent with the microscopy photos (Figure 4), which showed that the vessel elements of *Prosopis glandulosa* and *Acacia mearnsii* did not collapse and still allowed a sufficient capacity for water uptake. Throughout this procedure, *Acacia* samples showed weight increases varying from 56 to 90%, indicating a certain level of expansion. In other words, the wood, which became heavier and more compact, could not absorb much water unless the volume simultaneously expanded. The denser *Prosopis* samples exhibited lower water absorption capacities ranging from approximately 36% to 51% of its original dry weight. As with otherwise untreated wood, it is crucial to seal densified wood to prevent water uptake, especially under wet environmental conditions.

Lastly, in order to investigate the national electrical utility’s concerns regarding high-voltage electrical equipment placed on “non-conducting” wood, a series of electrical resistivity tests was conducted as part of this study (refer to Table 1). Plates of 1 cm^2^ and with a thickness of 1 mm were used in these measurements. Resistivities were tested using potential differences of up to 5000 V. In all instances and as anticipated, electrical resistance decreased by orders of magnitude as the voltage increased five-fold. For instance, at 1000 V, the resistance of the 80 min DL sample, which was initially 34 GΩ (giga-ohm), decreased to 1 GΩ at 5000 V.

As for increased sample density, in general, no significant impact was seen on electrical resistance in the case of otherwise (Std) untreated samples. Only in the pre-dried and oiled samples were definite and similar trends observed; at 5000 V, resistivities systematically decreased by roughly 50% with increased wood density. It is thus concluded that atmospheric moisture uptake in the Std samples plays a significant role in its resistance to flow of electrical current. Resistivities in all samples where moisture was removed or excluded were so high that readings could only be obtained at 5000 V and with very high values ranging from 170 to 463 GΩ.

For the purpose of comparison, measurements were conducted on several well-known insulating materials under similar (Std) conditions, namely Perspex (polymethylmethacrylate), PVC (polyvinylchloride), Teflon (polytetrafluoroethylene), borosilicate glass, and white paper (primarily composed of cellulose, either dried or oiled). Paper (even when oiled) performed worst in terms of electrical insulating capacity (2–3 GΩ) compared to all the other reference materials, which measured roughly similar (201–385 GΩ) to the various dried and oiled wood samples.

Electrical insulators typically possess strongly bonded electrons and a limited number of mobile charge carriers, resulting in high resistance and low conductivity. Applying a potential difference to an insulator allows for the determination of the electric field based on the voltage gradient across the material. Variations in the form or composition of the insulator lead to distinct voltage gradients. Electrical breakdown occurs when the electric field in a region exceeds the dielectric strength, causing the area to become conductive. This initiates further degradation until a conductive pathway is formed. The voltage at which the initial breakdown takes place is known as the breakdown voltage of the insulator material [57,58,59,60,61]. As shown in Table 1, voltage breakdowns only occurred with two of the *Prosopis* samples and paper at 5000 V.

## 4. Conclusions

This study presents comprehensive experimental data on the densification, machining behavior, and physicochemical properties of two invasive South African hardwood species, *Prosopis glandulosa* and *Acacia mearnsii*. Partial delignification using NaOH/Na_2_SO_3_ removed approximately 13–18% of the lignin in *Prosopis* and 23–25% in *Acacia*, which was followed by hot pressing at 5 MPa and 100 °C.

Densification resulted in substantial thickness reductions, with *Prosopis* compressed to 60% and *Acacia* to 50% of their original thickness after 80 min of delignification. Correspondingly, mechanical properties improved significantly. Flexural strength increased by 216% for *Prosopis* (to 22.61 MPa) and 334% for *Acacia* (to 24.65 MPa), while compressive strength increased by 253% for *Prosopis* and 231% for *Acacia*.

Nail resistance increased by 120%, from 2.94 MPa to 6.37 MPa in the most dense samples, indicating enhanced surface durability. Thermal degradation behavior showed three distinct stages, with the major decomposition occurring between 240 and 410 °C for *Prosopis* and between 240 and 350 °C for *Acacia*. Electrical resistivity of dried and oiled densified samples ranged from 170 to 463 GΩ at 5000 V, comparable to that of conventional insulating polymers, highlighting their potential as electrical insulating materials.

Despite densification, water absorption remained high (36–51% for *Prosopis* and 56–90% for *Acacia*), indicating that surface sealing is required for moisture-exposed applications. Flame-holding capacity decreased with increasing density, suggesting improved resistance to ignition, although dense samples may burn longer due to higher mass.

Overall, densification substantially enhanced mechanical performance, particularly for *Acacia*, while *Prosopis* showed more moderate improvements due to its higher initial density. The results demonstrate that invasive woody biomass can be transformed into high-performance engineered materials suitable for structural, furniture, and electrical insulation applications. Species-specific responses highlight the need for tailored processing conditions. Wood species differ in their ability to be densified (compressed) mainly because of differences in their anatomy, cell wall chemistry, moisture behavior, and microstructure. Low-density woods densify better because there is more space to collapse, while high-density woods already have thick walls, limited void space, and resist further compression. This is the reason why species like pine densify much more dramatically than very dense hardwoods, as will be illustrated in our next publications.

Large-scale production faces some hurdles, including high energy consumption during prolonged compression (often 24 h), challenges in chemical recovery of delignification agents, and the need for continuous processing methods to handle varying wood species as current batch processes limit industrial scalability. Long-term durability issues are also to be considered: despite gains in compressive strength and stiffness, densified wood exhibits moisture sensitivity, leading to the “springback” or set-recovery effect under cyclic humidity changes where absorbed water acts as a plasticizer, weakening hydrogen bonds and causing dimensional instability, swelling, or reduced load-bearing capacities. Weather resistance remains a concern for outdoor applications due to vulnerabilities to UV radiation, fungal decay, and termite attacks unless further treated, and thermal instability at elevated temperatures can induce creep or degradation, necessitating advanced post-treatments like lignin regeneration or hydrophobic coatings to ensure reliability in real-world cyclic environments. Overall, ongoing research is essential to optimize these woods for commercial viability without compromising sustainability [9,62,63,64].

## Figures and Tables

**Table 1:** Electrical resistance through 1 mm thick samples of *Prosopis glandulosa* (dark gray columns), *Acacia mearnsii* (light gray columns) as is, pre-dried, and oiled. Resistivities of reference materials were also measured under similar experimental conditions (bottom). Ambient temperature—ca 14 °C. Relative humidity—ca 50%. STD—sample as is. ∞—resistance too high to measure. Arc—resistance collapse.

Samples	Voltage (V)	Std (GΩ)		Dried (GΩ)		Oiled (GΩ)	
Untreated	500 1000 2500 5000	∞ ∞ 72 28	∞ 25 5 1.4	∞ ∞ ∞ 388	∞ ∞ ∞ 463	∞ ∞ ∞ 223	∞ ∞ ∞ 301
Cold pressed	500 1000 2500 5000	∞ ∞ 28 Arc	∞ 33 7 3.5	∞ ∞ ∞ 283	∞ ∞ ∞ 281	∞ ∞ ∞ 255	∞ ∞ ∞ 223
Hot pressed	500 1000 2500 5000	∞ ∞ 27 Arc	∞ 38 7 2	∞ ∞ ∞ 273	∞ ∞ ∞ 197	∞ ∞ ∞ 208	∞ ∞ ∞ 173
Boiled water	500 1000 2500 5000	∞ ∞ 11 4	∞ 34 5 1	∞ ∞ ∞ 221	∞ ∞ ∞ 180	∞ ∞ ∞ 204	∞ ∞ ∞ 193
40 min DL	500 1000 2500 5000	∞ 31 3 0.5	∞ 31 6 1	∞ ∞ ∞ 215	∞ ∞ ∞ 171	∞ ∞ ∞ 183	∞ ∞ ∞ 170
80 min DL	500 1000 2500 5000	∞ 34 4 1	∞ 32 4 0.8	∞ ∞ ∞ 189	∞ ∞ ∞ 197	∞ ∞ ∞ 123	∞ ∞ ∞ 169

Perspex	2500	∞					
	5000	385					
PVC	2500	∞					
	5000	271					
Teflon	2500	∞					
	5000	361					
Borosilicate glass	2500	∞					
	5000	201					
Paper	2500	2					
	5000	Arc					
Oiled Paper	2500	3					
	5000	Arc					

## Data Availability

The raw data supporting the conclusions of this article will be made available by the authors on request.

## Supplementary Materials

The following supporting information can be downloaded at: https://www.mdpi.com/article/10.3390/ma19050954/s1, Table S1: Process times of various wood samples. DL—delignified.; Table S2: Weight changes during processing.; Table S3: Crack comparison during nail tests. pc—partial crack, nc—no crack, fc—full crack.; Table S4: Water absorption.
