# Supplementary material for: Densification and Mechanical Enhancement of Invasive South African Hardwoods: Prosopis glandulosa and Acacia mearnsii
Source: Materials (Basel). 2026 Mar 1;19(5):954. doi: 10.3390/ma19050954 (PMC12985863; doi:10.3390/ma19050954)
Supplement: Supplementary file 1 [file materials-19-00954-s001.zip › materials-4148036-supplementary.pdf]

Supporting Information

Densification and Mechanical Enhancement of Invasive South African Hardwoods: *Prosopis glandulosa* and *Acacia mearnsii*

Matin Naghizadeh[1], Marthie E. Niemand[2], Ernst H.G. Langner[1], Aimin S. Sivanda[2], Karel G. von Eschwege[1*]

[1] Department of Chemistry, PO Box 339, University of the Free State, Bloemfontein, 9300, South Africa;
[2] Centre for Environmental Management, PO Box 339, University of the Free State, Bloemfontein, 9300. South Africa

*Corresponding author Tel: +27-51-4012923. Fax: +27-51-4446384.
*Karel G. von Eschwege: https://orcid.org/0000-0001-8801-4703
*E-mail address: vEschwKG@ufs.ac.za

Table S1. Process times of various wood samples. DL – delignified.

Wood	Treatment	Treatment time (min)	Press time (h)	Thickness (mm)
Prosopis glandulosa	None	-	-	25
	Cold press	-	3	24
	Cold press	-	3	24
	Hot press	-	5	19
	Hot press	-	5	19
	Boil water	60	12	18
	Boil water	60	12	18
	40 min DL	40	12	16
	40 min DL	40	12	16
	80 min DL	80	12	15
	80 min DL	80	12	15
Acacia mearnsii	None	-	-	25
	Cold press	-	3	24
	Cold press	-	3	24
	Hot press	-	5	19
	Hot press	-	5	19
	Boil water	60	12	16
	Boil water	60	12	15
	40 min DL	40	12	12
	40 min DL	40	12	12
	80 min DL	80	12	12
	80 min DL	80	12	13

Table S2. Weight changes during processing.

Wood name	Treatment	Wood blocks (g)	Before press (g)	After press (g)
Prosopis glandulosa	None	70.65	-	-
	Cold press	60.89	-	60.77
	Cold press	58.89	-	58.71
	Hot press	55.94	-	53.38
	Hot press	62.94	-	57.89
	Boil water	65.30	64.78	53.19
	Boil water	63.66	62.05	51.73
	40 min DL	62.19	66.76	48.78
	40 min DL	60.86	62.03	50.99
	80 min DL	60.67	67.62	50.05
	80 min DL	61.13	69.54	51.65
Acacia mearnsii	None	38.11	-	-
	Cold press	39.52	-	39.52
	Cold press	37.39	-	37.35
	Hot press	41.09	-	38.92
	Hot press	38.93	-	36.29
	Boil water	43.29	52.27	41.01
	Boil water	37.90	43.60	36.46
	40 min DL	39.05	54.75	35.14
	40 min DL	37.90	50.61	35.49
	80 min DL	38.02	49.26	35.46
	80 min DL	39.72	49.35	36.89

Table S3. Crack comparison during nail tests. pc – partial crack, nc – no crack, fc – full crack.

Wood	Treatment	Cracks
Prosopis glandulosa	None	nc
	Cold press	pc
	Cold press	pc
	Hot press	pc
	Hot press	pc
	Boil water	pc
	Boil water	pc
	40 min DL	pc
	40 min DL	nc
	80 min DL	pc
	80 min DL	pc
Acacia mearnsii	None	fc
	Cold press	fc
	Cold press	nc
	Hot press	nc
	Hot press	fc
	Boil water	nc
	Boil water	fc
	40 min DL	nc
	40 min DL	fc
	80 min DL	nc
	80 min DL	fc

Table S4. Water absorption.

Wood name	Treatment	Before H_2O (g)	After H_2O (g)	Water absorption (%)
Prosopis glandulosa	None	0.3464	0.4855	40.16
	Cold press	0.3923	0.549	39.94
	Cold press	0.3495	0.4743	35.71
	Hot press	0.302	0.4425	46.52
	Hot press	0.5584	0.8238	47.53
	Boil water	0.4863	0.6918	42.26
	Boil water	0.5301	0.7413	39.84
	40 min DL	0.5646	0.8399	48.76
	40 min DL	0.4453	0.6729	51.11
	80 min DL	0.4402	0.6600	49.93
	80 min DL	0.6727	0.9798	45.65
Acacia mearnsii	None	0.4431	0.7073	59.63
	Cold press	0.4517	0.7832	73.39
	Cold press	0.3448	0.5874	70.36
	Hot press	0.3859	0.6085	57.68
	Hot press	0.4791	0.8165	70.42
	Boil water	0.5468	0.855	56.36
	Boil water	0.4173	0.7942	90.32
	40 min DL	0.4794	0.7439	55.17
	40 min DL	0.662	1.1783	77.99
	80 min DL	0.5693	0.9415	65.38
	80 min DL	0.4265	0.6641	55.71
